# Single-Nucleotide Polymorphisms in *XPO5* are Associated with Noise-Induced Hearing Loss in a Chinese Population

**DOI:** 10.1155/2020/9589310

**Published:** 2020-02-17

**Authors:** Ning Wang, Boshen Wang, Jiadi Guo, Suhao Zhang, Lei Han, Juan Zhang, Baoli Zhu

**Affiliations:** ^1^Key Laboratory of Environmental Medicine Engineering of Ministry of Education, School of Public Health, Southeast University, Nanjing 210009, Jiangsu, China; ^2^Jiangsu ProvincialCenter for Disease Prevention and Control, Nanjing 210009, Jiangsu, China; ^3^Center for Global Health, School of Public Health, Nanjing Medical University, Nanjing 210000, Jiangsu, China; ^4^School of Public Health, NantongUniversity, Nantong 226000, Jiangsu, China

## Abstract

**Objectives:**

The purpose of this study was to investigate the correlation between single-nucleotide polymorphism (SNP) in 3′UTR of *XPO5* gene and the occurrence of noise-induced hearing loss (NIHL), and to further explore the regulatory mechanism of miRNAs in NIHL on *XPO5* gene and the occurrence of noise-induced hearing loss (NIHL), and to further explore the regulatory mechanism of miRNAs in NIHL on

**Methods:**

We conducted a case-control study involving 1040 cases and 1060 controls. The effects of SNPs on *XPO5* gene and the occurrence of noise-induced hearing loss (NIHL), and to further explore the regulatory mechanism of miRNAs in NIHL on

**Results:**

We genotyped four SNPs (rs2257082, rs11077, rs7755135, and rs1106841) in the *XPO5* gene and the occurrence of noise-induced hearing loss (NIHL), and to further explore the regulatory mechanism of miRNAs in NIHL on *XPO5* gene and the occurrence of noise-induced hearing loss (NIHL), and to further explore the regulatory mechanism of miRNAs in NIHL on *XPO5* gene and the occurrence of noise-induced hearing loss (NIHL), and to further explore the regulatory mechanism of miRNAs in NIHL on *XPO5. Conclusion*. The genetic polymorphism, rs11077, within *XPO5* is associated with the risk of noise-induced hearing loss in a Chinese population.*XPO5* gene and the occurrence of noise-induced hearing loss (NIHL), and to further explore the regulatory mechanism of miRNAs in NIHL on

## 1. Introduction

Noise-induced hearing loss (NIHL) refers to progressive sensorineural hearing impairment caused by patients exposed to a noisy environment. NIHL has become a major public health problem with industrialization, the increase in social noise, and the prolongation of life expectancy. Based on the global disease burden report issued by the WHO in 2005, occupational noise-associated hearing loss accounts for 16% of adult hearing loss worldwide, which is about 4 million disability-adjusted life years (DALYs) [1]. There are greater than 10 million workers employed in high-noise environments in China, of which at least 10% have different levels of hearing loss. Although environmental factors play a vital part in the development of NIHL, population epidemiologic studies have shown that, with the exception of the influence of other confounding factors, hereditary factors account for up to 50% of the variability in hearing loss after noise exposure [2,3].

MicroRNAs (miRNAs) are highly-conserved, endogenous noncoding single-stranded RNAs with posttranscriptional regulatory functions that are found in eukaryotes and consist of approximately 22 nucleotides (nt) [4,5]. Greater than 700 kinds of miRNAs have been identified in humans and regulate at least 30% of protein-coding gene expression [6,7]. miRNAs are transcribed into primary transcripts of miRNAs (pri-miRNAs) in the nucleus by RNA polymerase II and then further assimilated by RNase III *Drosha* to form a hairpin structure of approximately 60- to 70-nt precursor miRNAs (pre-miRNAs) [8,9]. Pre-miRNAs are transported from the nucleus to the cytoplasm under the synergistic action of transport receptor exportin-5 (*XPO5*). It is believed that single-nucleotide polymorphisms (SNPs) exist in the binding sites of the gene encoding miRNAs and related target genes, which can directly or indirectly affect gene expression and protein function [10].

Recent studies have confirmed that the abnormal expression of miRNAs is related to many diseases, including auditory diseases [11]. There is increasing evidence that the imbalance of miRNAs in NIHL affects the expression of target genes and further affects the necessary cellular processes, including cell metabolism, proliferation, differentiation, and apoptosis [12,13]. Compared with the control group, the expression of miRNA-24, miRNA-185-5p, and miRNA-451a increased significantly in the NIHL group [14]. Li et al. [15] reported that the serum miRNA-1229-5p level of male workers suffering from NIHL was significantly higher than the control group.

*XPO5* exists in the nuclear membrane and participates in the transport of pre-miRNAs. Previous results have indicated that overexpression of *XPO5* enhances the activity of miRNAs, and under- or nonexpression of *XPO5* results in a decrease in the nuclear output of pre-miRNAs [16,17]. SNPs associated with miRNAs in the *XPO5* 3′untranslated region (3′UTR) affect the risk of disease in the synthesis pathway of miRNAs [18]. Currently, there is a lack of research on the relationship between*XPO5* and the risk of NIHL development; however, several studies have demonstrated that the transporter, *XPO5,* is involved in the miRNA pathway. The structural changes of *XPO5* may cause abnormal expression of the miRNA, leading to tumorigenesis [19–21], which may also be the case in the hearing system. Based on bioinformatics prediction and statistical analysis, we found that SNPs in *XPO5* may play a potential role in NIHL. In this study, we focused on the SNPs located in the binding region of miRNAs and *XPO5*. For significant SNPs, we performed functional validation to evaluate the potential function of these SNPs on *XPO5*.

## 2. Materials and Methods

### 2.1. Study Population Collection

In the current study, a total of 1040 cases and 1060 controls were recruited from the Yizheng Branch of the SAIC Volkswagen Automobile Co., Ltd. The subjects were engaged in steady-state noise work for a long time, and the exposure period of noise was not less than 1year. Workers exposed to noise did not have any history of disease or current illness that might affect their hearing, nor of the long-term use of ototoxic drugs. The definition of hearing loss was as follows: the subject's audiogram falls at high frequencies; the average hearing threshold of high frequencies in both ears and the better unilateral ear are >25 dB; and the high frequencies are greater than the low frequencies. Normal hearing refers to a subject's high and low frequency threshold ≤25 dB. NIHL cases and controls were matched, including age, gender, smoking status, and noise exposure time. This study was approved by the Ethics Committee of Jiangsu Centers for Disease Control and Prevention, and all of the subjects signed the informed consent in person. Considering the use of data analysis, the private information of subjects involved in the study was encrypted.

### 2.2. SNP Selection

SNP loci located in the *XPO5* gene were found from NCBIdbSNP (http://www.ncbi.nlm.nih.gov/SNP) and ENSEMBL (http://www.ensembl.org/). The principles for screening SNPs are indicated as follows: (a) *XPO5* binding site; (b) minor allele frequency (MAF) > 0.05; (c) *p* of Hardy–Weinberg equilibrium (HWE) > 0.05; and (d) linkage disequilibrium (LD) of *r*^*2*^ >0.8. Four SNPs (rs2257082, rs11077, rs7755135, and rs1106841) were selected as candidate SNPs because the target gene (*XPO5*) was associated with the pathogenesis of NIHL.

### 2.3. Genetic Analysis

Peripheral blood samples from the subjects were stored in Vacutainers® containing the anticoagulant, (ethylenediaminetetraacetic acid) EDTA. The total DNA template was extracted using a DNA extraction kit (QIAGEN, Duesseldorf, Germany). The concentration and purity of the extracted DNA were determined using an ultraviolet spectrophotometer (NanoDrop ND-1000; Thermo Scientific, Wilmington, DE, USA). Based on the NCBIdbSNP database, the primers of the SNP locus were designed. The genomic DNA was amplified using an ABI7900HT real-time PCR system (Applied Biosystems, Foster City, CA,USA) at 94°C for 5 min, followed by 40 cycles at 94°C for 20 s, 56°C for 30 s, 72°C for 30 s, and 72°C for 7 min. Detection of gene polymorphisms was controlled by ABITaqManSNP genotyping assays (Applied Biosystems, Foster City, CA, USA). Five percent samples were randomly sampled for quality control of duplicate genotyping, and the reproducibility of SNPs was 100%.

### 2.4. Plasmid Construction

Human miRNA-4763-5p, miRNA-5002-3p, and miRNA-617 were cloned into the expression vector pcDNA3.1(+) to generate stably-transfected human embryonic kidney cell lines (HEK293T) using forward primers (GAATCTGGTCACCTGATGGGA) and reverse primers (GTGCCTGAGTGGACCTTGAG). The plasmid containing the sequence of the wild-type or mutant miRNA-4763-5p, miRNA-5002-3p, or miRNA-617 binding *XPO5*, was cloned into the luciferase reporter vector (pGL3-CMV-LUC-MCS). The successfully constructed expression vector was inoculated into LB (Luria–Bertani) medium and cultured on a shaking table (220 rpm)at 37°C for 24 h. The plasmids were extracted and sequenced using a high-purity plasmid extraction kit (QIAGEN).

### 2.5. Reagents, Cell Culture, and Transfection

The HEK293T cell lines used in this study were obtained from Novobio Scientific (Shanghai, China). All cells were preserved in Dulbecco's Modified Eagle's medium (DEME) fortified with 10% fetal bovine serum (FBS) and placed in a humidified atmosphere with 5% CO_2_ at 37°C. miRNA-4763-5p mimics, miRNA-5002-3p mimics, miRNA-617 mimics, and pRL-TK (internal reference) were purchased from Genomeditech (Shanghai, China). The transfection experiment was carried out when the cells reached 70%–80% confluence. The plasmid transfection was conducted on the basis of the instructions of the manufacturer of Lipofectamine 2000™ (Invitrogen, Carlsbad, CA, USA).

### 2.6. Luciferase Reporter Assay

For luciferase analysis, HEK293T cells were inoculated into 24-well plates. Cells transfected with luciferase vectors containing T wild-type or G-mutant *XPO5* fragments and miRNAs (miRNA-4763-5p, miRNA-5002-3p, and miRNA-617) were collected and cultured in medium and washed twice with phosphate-buffered saline (PBS). The cells were fully lysed by adding 100 *μ*l of 1× passive lysis buffer (PLB; Promega, Madison, WI, USA) to each well. Luciferase activity measurement was carried out on the basis of the operating instructions of the luciferase reporter assay system (Promega, Madison, WI, USA). The activity of Renilla luciferase was normalized to that of Firefly luciferase. All transfections were in triplicate.

### 2.7. Statistical Analysis

Frequencies of alleles and genotypes at SNP loci were obtained by direct counting. The association of genotype and gene frequency between the case and control groups was determined by a chi-square test. An unconditional univariate logistic regression model was used to analyze the odds ratios (ORs) and their 95% confidence intervals (95% CIs) and to evaluate the correlation between the genotype of miR-SNPs and the occurrence of NIHL. Values are shown as the mean ± standard deviation. Differences were analyzed by SPSS (version 19; IBM, Armonk, NY, USA), and correction of the haplotype *p* value (Pc) was done using the Sidak, Holm's correction, and the results were regarded as statistically significant at *p* < 0.05.

## 3. Results

### 3.1. Characteristics and Clinical Features of Study Participants

A total of 2100 participants were recruited (1040 cases and 1060 controls). Detailed demographic data of the individuals are shown in Table 1. Controls were matched with NIHL cases in the distribution of age (*p*=0.537), gender (*p*=0.074), cigarette smoking (*p*=0.448), alcohol consumption (*p*=0.374), duration of noise exposure (*p*=0.511), and intensity of noise exposure (*p*=0.325). A higher high-frequency hearing threshold shift was observed in cases ((40.73 ± 13.40) dB) compared to controls (16.63 ± 4.75 dB). There were significant differences in the high-frequency hearing threshold between the two groups (*p* < 0.001).

### 3.2. Association between the XPO5 Gene Polymorphism and NIHL Susceptibility

The characteristics of selected SNPs with an MAF >0.05 are listed in Table 2. The *XPO5* loci rs2257082, rs11077, rs7755135, and rs1106841 were consistent with the Hardy–Weinberg equilibrium (HWE; *p* > 0.05) in the control group; the minimum *p* value of HWE was 0.15. Multivariate logistic regression analysis showed that the rs2257082, rs11077, and rs7755135 loci, but not rs1106841, of the *XPO5* gene were significantly associated with NIHL after excluding the potential confounders (age, gender, cigarette smoking, and alcohol consumption, duration of noise exposure, and intensity of noise exposure). SPSS 19.0 was used for statistical analysis.

As illustrated in Table 3, the rs2257082 AG/GG carriers showed an increased risk of NIHL compared to the AA carriers in the dominant model (adjusted OR = 1.55, 95% CI: 1.23–1.95, *p* < 0.001). The rs11077TG/GG carriers had a significantly increased association with NIHL susceptibility than TT carriers in the dominant (adjusted OR = 1.93, 95% CI: 1.48–2.52, *p* < 0.001) and recessive models (adjusted OR = 3.03, 95% CI: 1.19–7.76, *p* < 0.001). There was a higher risk of NIHL in the *XPO5* gene rs7755135TT carriers than CC carriers in the codominant (adjusted OR = 2.70, 95% CI: 1.53–4.77, *p* < 0.001) and recessive models (adjusted OR = 2.42, 95% CI: 1.40–4.19, *p* < 0.001). No statistically significant correlation was obtained for SNPrs1106841 in any of the models (codominant, dominant, or recessive models). In the allele model, the rs11077G carriers (adjusted OR = 1.63, 95% CI: 1.30–2.03) had a significantly higher risk for NIHL (*p* < 0.001) and rs7755135T carriers (adjusted OR = 1.12, 95% CI: 1.01–1.39) had a significantly higher risk for NIHL (*p*=0.037). The results indicated that *XPO5*SNPrs2257082, rs11077, and rs7755135 may have a connection to NIHL.

### 3.3. Analysis of High-Frequency Hearing Threshold Shift (HFTS) in Selected SNPs

**Figure 1:**
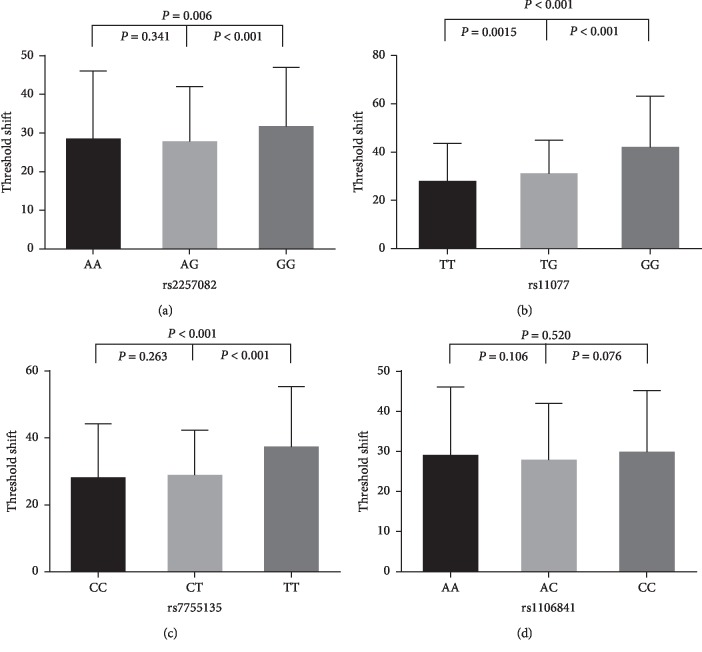
Comparison of high-frequency hearing threshold shift of four SNPs. Comparison of high-frequency hearing threshold shift of rs2257082, rs11077, rs7755135, and rs1106841 genotypes in all subjects. Data are presented as mean ± SD, followed by analysis by ANOV. NS: no statistical significance.

The data of all subjects in Figure 1 described that the HFTS of the *XPO5*rs2257082 AA genotype was mainly in the range of 28.48 ± 17.62 dB, and those of the AG and GG genotypes were in the range of 27.76 ± 14.24 dB and 31.64 ± 15.34 dB, respectively. The HFTS in the GG genotypes of rs2257082 were significantly higher than in the AA genotypes (*p*=0.006). For rs11077, the TT genotype was mainly in the range of 27.96 ± 15.77 dB, and the TG and GG genotypes were 30.96 ± 13.98 dB and 41.91 ± 21.16 dB, respectively. The HFTS in the rs11077GG and TG genotype subjects were significantly higher than in the TT genotypes (*p* < 0.001and *p*=0.0015, respectively). The rs7755135 CC genotype was mainly in the range of 28.05 ± 16.28 dB, while the CT and TT genotypes were in the range of 28.90 ± 13.47 dB and 37.27 ± 18.12 dB, respectively. The TT genotype exhibited a significantly greater HFTS risk compared with the CC and CT genotypes (*p* < 0.001 and *p* < 0.001, respectively); however, the HFTS of the AA, AC, and CC genotypes of rs1106841 were mainly 29.01 ± 17.10 dB, 27.85 ± 14.13 dB, and 29.87 ± 15.31 dB, respectively. There were no significant differences in HFTS among the AA, AC, and CC genotypes (*p*=0.106, *p*=0.076, and *p*=0.520, respectively).

### 3.4. Analysis of SNP (rs2257082, rs11077, rs7755135, and rs1106841) Haplotype Distribution

**Figure 2:**
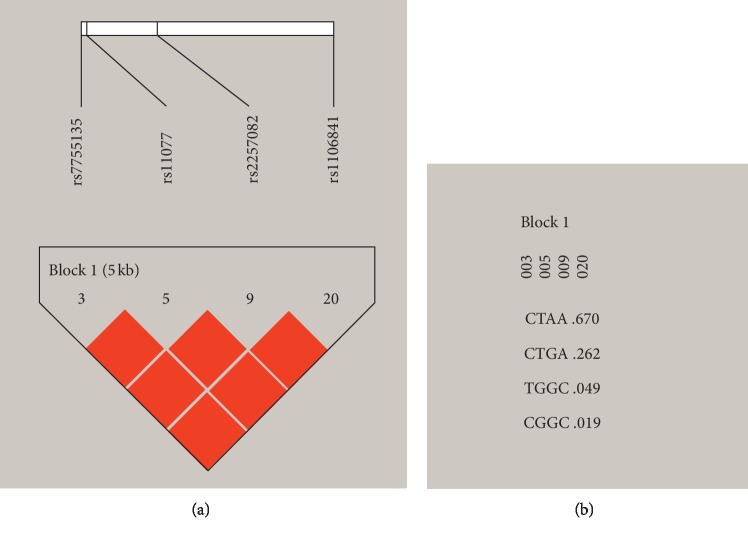
Reconstructed linkage disequilibrium (LD) plot for the four single-nucleotide polymorphisms (SNPs).

Figure 2 showed that the linkage disequilibrium of *XPO5*rs2257082, rs11077, rs7755135, and rs1106841.  Table 4 summarizes the haplotype frequencies of SNPs analyzed between NIHL cases and control groups. Five common haplotypes (frequency >3%) were selected from four SNPs, which accounted for 90% of haplotype variation. The GGTA and GTCC haplotypes (rs2257082-rs11077-rs7755135-rs1106841) were associated with an increased risk of NIHL (OR = 1.54, 95% CI: 1.30–1.1.94, *p* < 0.001; OR = 1.46, 95% CI: 1.18–1.81, *p* < 0.001). The GTCA haplotype was associated with a decreased risk of NIHL (OR = 0.81, 95% CI: 0.68–0.96, *p*=0.02).

### 3.5. Predicted miRNAs That Potentially Bind to *XPO5*rs11077

**Figure 3:**
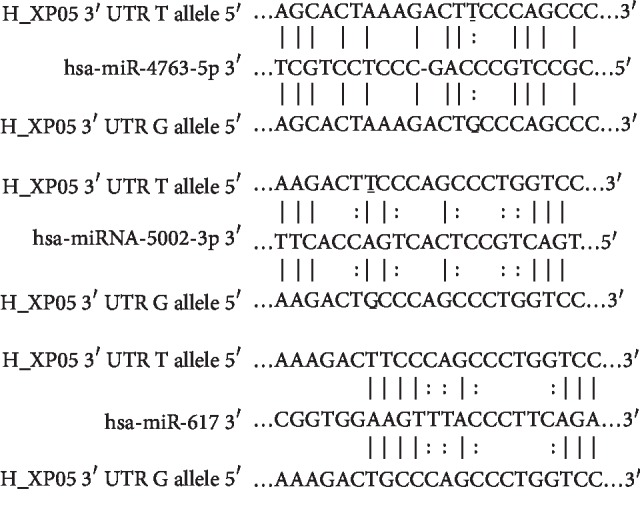
Predicted binding of the candidate miRNAs (miR-4763-5p, miRNA-5002-3p, and miR-617) to SNPrs11077 of *XPO5*3′UTR.

To demonstrate whether or not the rs11077SNP affects the prediction of miRNA binding to *XPO5*, we performed a bioinformatics analysis of *XPO5*. The results indicated that *XPO5*rs11077 is located in a potential binding region for incorporation of miRNAs, including miRNA-4763-5p, miRNA-5002-3p, and miRNA-617. *XPO5* contains the binding site of predicted miRNAs, as shown in Figure 3.

### 3.6. SNPs Interfered with the Interaction between miRNA (miRNA-4763-5p, miRNA-5002-3p, and miRNA-617) and *XPO5*

**Figure 4:**
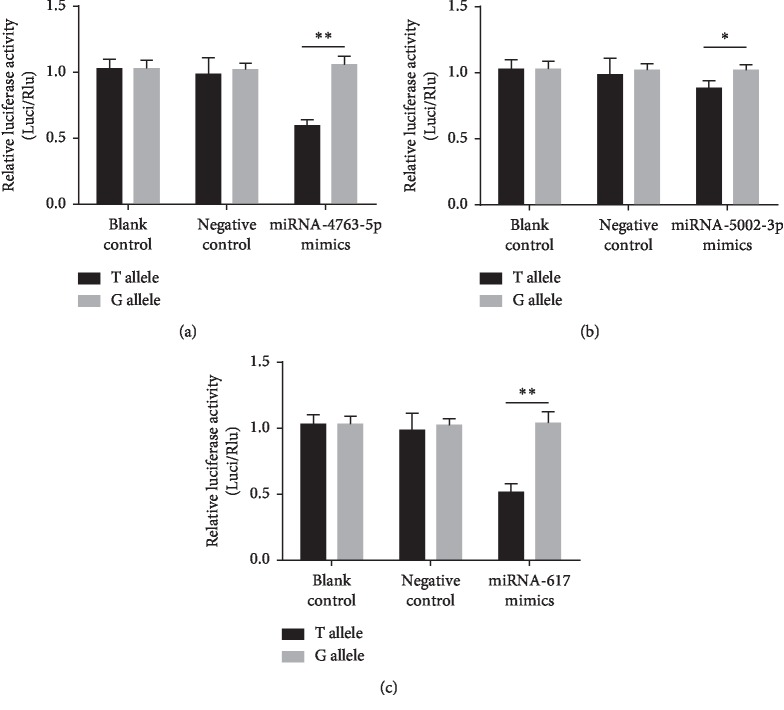
SNPrs11077T or G allele in the 3′-UTR of *XPO5* differences with the presence or interference of miRNAs (miR-4763-5p, miRNA-5002-3p, and miR-617) was analyzed by dual-luciferase report assay. Negative control means adding an unrelated sequence of miRNAs to the pGL3 vector coupled to the *XPO5*3′-UTR regions (pGL3-3′UTR). Relative luciferase activity was expressed by the ratio of Firefly/Renilla activity. Data were presented as the mean ± SD. ^*∗*^*p* < 0.05 and ^*∗∗*^*p* < 0.01.

Transient transfection was carried out *in vitro*, and the expression of related activities was analyzed and measured by the dual-luciferase reporting system to illustrate whether or not the SNPrs11077T>G variant affected the binding of *XPO5* to miRNAs (miRNA-4763-5p, miRNA-5002-3p, and miRNA-617).  Figure 4 indicates that constructs containing the G allele of *XPO5* combined with miRNA-4763-5p, miRNA-5002-3p, and miRNA-617 mimics significantly increased luciferase activity compared with the T allele in HEK293T (*p* < 0.01, *p* < 0.05, and *p* < 0.01, respectively). These data suggested that miRNA-4763-5p, miRNA-5002-3p, and miRNA-617 may directly target *XPO5* with the rs11077G allele.

## 4. Discussion

NIHL is one of the most common occupational diseases that seriously affect human health [11]. Globally, the incidence of NIHL is on the rise. The pathogenesis of NIHL has not been fully clarified [22]. An etiologic investigation showed that chronic ear diseases, alcohol consumption, smoking, and occupational factors are related to the occurrence and development of NIHL, but different individuals vary in their sensitivity to individual causes.

Currently, studies on miRNAs and hearing loss have demonstrated that these miRNAs can be used as potential biomarkers to indicate NIHL [15,23]. In a study involving miRNA-15a-1 and miRNA-18a in the development of the ear structure of zebrafish, it was found that the number of hair cells in the deformed body decreased, and the inner ear structure was abnormal [24]. MiR-34 mediates hearing impairment associated with cell death in the inner ear of a mouse model [25]. *XPO5* is related to the nuclear output of miRNAs [26]. A synergistic effect exists between Ran-GTP and transport receptor *XPO5*, which transports pre-miRNAs from the nucleus to the cytoplasm [27,28]. After digestion and double helix unwinding, pre-miRNAs bind with the RNA-induced silencing complex (RISC), which contains GEMIN3 and GEMIN4, to synthesize RISC-miRNA complexes [20]. The binding of RISC to the specific sequence of the 3′UTR of target mRNAs results in the inhibition of the cleavage or translation of the mRNA, which interferes with the protein synthesis of the target gene at the posttranscriptional level [29,30]. Reducing the expression of *XPO5* can decrease the expression of miRNAs, which may lead to the occurrence and development of hearing loss.

Considering the effect of age on hearing, we matched people in our study. Mizoue et al. [31] have proved that smoking can increase the risk of NIHL. To eliminate the interference of cigarette smoking, the matching included cigarette smoking. When binding of miRNAs to target sequences occurs at or near the miRNA junction in the 3′UTR, the SNPs have an effect by establishing or eliminating the binding sites of miRNAs in target genes, thus losing the original regulatory function and producing significant genetic effects. Accordingly, we systematically investigated the potential correlation between the genetic polymorphism of *XPO5* and NIHL in the Chinese population and discovered SNPs (rs2257082, rs11077, and rs7755135) in the *XPO5* gene. Our data showed that the frequencies of the rs2257082GG, rs11077GG, and rs7755135TT of the *XPO5* gene were significantly increased in NIHL cases compared to the control group. Therefore, we speculated that the rs2257082, rs11077, and rs7755135 loci SNPs of the *XPO5* were associated with NIHL risk. Haploid analysis revealed that the GGTA and GTCC haplotypes (rs2257082-rs11077-rs7755135-rs1106841) increased the risk of NIHL, and the GTCA haplotype was associated with a decreased risk of NIHL. The results support our hypothesis that *XPO5* polymorphism may be related to NIHL susceptibility.

miRNAs can degrade or inhibit protein translation by means of complete or incomplete complementary pairing with target gene mRNA. Therefore, miRNAs play a significant role in posttranscriptional regulation of gene expression. The mutation of the binding site of the target gene of miRNAs will affect the biosynthesis or biological function of miRNAs, which will lead to the disorder of cellular function and eventually result in the occurrence of diseases. SNPs can have a profound impact on miRNA function, including transcription, maturation, and target specificity [32], and it can also affect the occurrence of NIHL [33]. Recent studies have shown that SNPrs11077 in *XPO5* gene is related to the risk of esophageal cancer, colorectal cancer, and renal cancer [34–36]. The A>C polymorphism of *XPO5* gene will reduce the risk of CAD (coronary artery disease), which may be due to the influence on the expression of mature miRNAs and their gene function [37]. At the same time, functional SNPs in miRNA biogenetic pathway genes have been confirmed to be related to the increased risk of NIHL [33,38]. Based on all the above studies, we analyzed the relationship between the functional sites of *XPO5* of miRNA processing gene and NIHL, and its effect on miRNA expression. In this study, luciferase reporter assays preliminarily verified whether miRNA interacted with target gene *XPO5*3′UTR and further determined the site of interaction between miRNA and target gene *XPO5*3′UTR.

In addition to the interaction of multiple factors, rs11077 had the most significant correlation with NIHL compared with other loci. We selected rs11077 for functional verification. By virtue of the mutations located in the secondary structure of miRNA, as well as the number of mutants that can be detected, we investigated the possible effects of these mutations on target genes. First, we predicted potential targets based on bioinformatics programs (TargetScan, Microinspector, and miRanda). Meanwhile, considering the complementation, evolutionary conservation, accessibility, and thermal stability of the target gene locus (rs11077) to miRNA, we included these miRNAs (miRNA-4763-5p, miRNA-5002-3p, and miRNA-617) and conduct subsequent studies on luciferase activity. These miRNAs contain binding sites that match the seed region of *XPO5* perfectly. Importantly, miRNA-4763 has recently been shown to contribute specifically to multidrug resistance in human cancer cells [32]. Wang et al. [33] reported that downregulation of miRNA-4763-3p expression increased the susceptibility to gastric cancer by targeting MDR. It was also predicted that the potential regulatory pathway of *XPO5*rs11077, and the binding ability of miRNA-4763-5p/miRNA-5002-3p would be affected when the T wild-type allele mutated into the G allele [34]. The results of luciferase reporter gene activity analysis in our study showed that the translation level of luciferase-UTR was controlled by miRNA-4763-5p, miRNA-5002-3p, and miRNA-617 compared with the T-allele miRNA-4763-5p and the miRNA-617, the G allele resulted in increased luciferase expression. This finding indicated that the mutation allele of rs11077 affected the binding of miRNAs (miRNA-4763-5p, miRNA-5002-3p, and miRNA-617) to *XPO5*.

## 5. Conclusion

In summary, our study provides evidence for the first time that the SNPrs11077G allele and the haplotype (rs2257082, rs11077, rs7755135, and rs1106841) had an association with the risk of NIHL in a Chinese population. It was also verified that the regulation of *XPO5* expression by miRNA-4763-5p, miRNA-5002-3p, and miRNA-617 might be achieved by SNPrs11077.

## Figures and Tables

**Table 1:** Demographic characteristics and clinical features.

Variables	Cases (*n* = 1040)		Controls (*n* = 1060)		*p*
	*n*	%	*n*	%	
Age (years), mean ± SD	39.50 ± 8.52		39.27 ± 8.34		0.537
Sex					0.074
Male	978	94.04	980	92.45	
Female	62	5.96	80	7.55	
Smoking					0.448
Now	533	52.46	515	49.71	
Ever	68	6.69	76	7.34	
Never	415	40.85	445	42.95	
Drinking					0.374
Now	229	22.76	265	25.33	
Ever	214	21.27	221	21.13	
Never	563	55.96	560	53.54	
Work time with noise (years), mean ± SD	16.91 ± 9.57		16.64 ± 9.23		0.511
Exposure level with noise (dB), mean ± SD	87.55 ± 14.98		86.89 ± 15.74		0.325
High-frequency hearing threshold (dB), mean ± SD	40.73 ± 13.40		16.63 ± 4.75		<0.001

**Table 2:** General information of selected SNPs and Hardy–Weinberg test.

SNP	Chromosome	Functional consequence	MAF			Regulome DB	
			Control	Database^a^	*p* ^*b*^	Function annotation	Score
rs2257082	6 : 43492578	Synonymous variant	0.97	0.32	0.75	Protein binding; chromatin structure; histone modifications	4
rs11077	6 : 43490947	3 prime UTR variant	0.73	0.40	0.15	Protein binding; chromatin structure; histone modifications	4
rs7755135	6 : 43490809	3 prime UTR variant	0.67	0.22	0.61	Protein binding; single nucleotides; chromatin structure; histone modifications	1f
rs1106841	6 : 43496662	Splice region variant	0.87	0.39	0.88	Motifs; chromatin structure; histone modifications	5

^a^Data from NCBIdbSNP. ^*b*^*p* value of Hardy–Weinberg test.

**Table 3:** Distribution of four polymorphisms and the association with NIHL.

Genetic models	Genotypes	Cases *n* = 1040	Controls *n* = 1060	*p* ^a^	Adjusted OR (95% CI)^a^	Holm	SidakSS	SidakSD
*rs2257082*		*n* = 1030	*n* = 1053					

Codominant	AA	324	380		1.00 (ref)			
	AG	546	515	2.69*E* − 4	1.54 (1.22–1.94)			
	GG	160	158	1.51*E* − 3	1.73 (1.23–2.44)			

Dominant	AA	324	380		1.00 (ref)			
	AG/GG	706	673	2.09*E* − 4	1.55 (1.23–1.95)			

Recessive	AA/AG	870	895		1.00 (ref)			
	GG	160	158	0.26	1.16 (0.89–1.51)			

Alleles	A	1194	1275		1.00 (ref)			
	G	866	831	0.09	1.11 (0.98–1.26)	0.18	0.314	0.172
*rs11077*		*n* = 1036	*n* = 1056					

Codominant	TT	835	921		1.00 (ref)			
	TG	186	129	8*E* − 6	1.85 (1.41–2.43)			
	GG	15	6	7.1*E* − 3	3.66 (1.42–9.41)			

Dominant	TT	835	921		1.00 (ref)			
	TG/GG	201	135	1*E* − 6	1.93 (1.48–2.52)			

Recessive	TT/TG	1021	1050		1.00 (ref)			
	GG	15	6	2.04*E* − 2	3.03 (1.19–7.76)			

Alleles	T	1856	1971		1.00 (ref)			
	G	216	141	1.43*E* − 5	1.63 (1.30–2.03)	5.71*E* − 5	5.71*E* − 5	5.71*E* − 5
*rs7755135*		*n* = 1034	*n* = 1057					

Codominant	CC	689	735		1.00 (ref)			
	CT	305	302	0.12	1.19(0.95–1.49)			
	TT	40	20	6.19*E* − 4	2.70(1.53–4.77)			

Dominant	CC	689	735		1.00 (ref)			
	CT/TT	345	322	0.04	1.26(1.01–1.57)			

Recessive	CC/CT	994	1037		1.00 (ref)			
	TT	40	20	1.65*E* − 3	2.42(1.40–4.19)			

Alleles	C	1683	1772		1.00 (ref)			
	T	385	342	0.037	1.12 (1.01–1.39)	0.112	0.141	0.108
*rs1106841*		*n* = 1035	*n* = 1052					

Codominant	AA	467	491		1.00 (ref)			
	AC	483	463	0.18	1.16(0.93–1.44)			
	CC	85	98	0.88	0.97(0.67–1.40)			

Dominant	AA	467	491		1.00 (ref)			
	AC/CC	568	561	0.22	1.15(0.92–1.42)			

Recessive	AA/AC	950	954		1.00 (ref)			
	CC	85	98	0.35	0.86(0.62–1.18)			

Alleles	A	1417	1445		1.00 (ref)			
	C	653	659	0.88	1.01 (0.89–1.15)	0.875	0.999	0.875

^a^Adjusted for age, sex, smoking, and drinking in the logistic regression model.

**Table 4:** Frequencies of inferred haplotypes among the cases and controls and their association with risk NIHL.

Haplotypes^a^	Case (freq)	Control (freq)	Chi2	Pearson's *p*	OR (95% CI)	Holm	SidakSS	SidakSD	Global *p* value
GGTA	199 (0.10)	136 (0.07)	14.21	1.63*E* − 04	1.54 (1.30–1.94)	8.16*E* − 04	8.16*E* − 04	8.16*E* − 04	1.55*E* − 06
GTTA	166 (0.08)	196 (0.09)	2.13	0.14	0.85 (0.69–1.06)	0.17	0.54	0.17	
GTCA	261 (0.13)	320 (0.15)	5.71	0.02	0.81 (0.68–0.96)	0.05	0.08	0.05	
GTCC	222 (0.11)	160 (0.08)	12.41	4.28*E* − 04	1.46 (1.18–1.81)	0.001	0.002	0.001	
ATCC	1186 (0.58)	1264 (0.61)	2.93	0.09	0.90 (0.79–1.02)	0.17	0.37	0.17	

^a^The alleles of the haplotypes were arrayed as rs2257082-rs11077-rs7755135-rs1106841. Haplotypes with frequency <0.03 are ignored.

## Data Availability

The data sets supporting the results of this article are included within the article.
