# Erratum to “Single-Nucleotide Polymorphisms in XPO5 are Associated with Noise-Induced Hearing Loss in a Chinese Population”

**DOI:** 10.1155/2020/9649346

**Published:** 2020-12-21

**Authors:** Ning Wang, Boshen Wang, Jiadi Guo, Suhao Zhang, Lei Han, Juan Zhang, Baoli Zhu

**Affiliations:** ^1^Key Laboratory of Environmental Medicine Engineering of Ministry of Education, School of Public Health, Southeast University, Nanjing 210009, Jiangsu, China; ^2^Jiangsu Provincial Center for Disease Prevention and Control, Nanjing 210009, Jiangsu, China; ^3^Center for Global Health, School of Public Health, Nanjing Medical University, Nanjing 210000, Jiangsu, China; ^4^School of Public Health, Nantong University, Nantong 226000, Jiangsu, China

In the article titled “Single-Nucleotide Polymorphisms in XPO5 are Associated with Noise-Induced Hearing Loss in a Chinese Population” [1], the information “Ning Wang, Boshen Wang contributed equally to this article” was omitted from the Authors' Contributions section. The corrected section appears below:
